# Notes on Preparations for the Sick

**Published:** 1893-12

**Authors:** 


### Notes on Preparations for the Sick.

**Cachets (Morstadt). Cachet Closing Apparatus.**—Thomas Christy and Co., London.—This apparatus appears to be as perfect as it is possible to be. Nicely made cachets are a luxury. If one has to take anything nauseous, surely this must be the way to do it. If, however, the nauseous powder adheres to the outside, from breakage and discharge of the contents of one, then the result is by no means satisfactory. These cachets are the most capacious we have seen, the double hat shape being a great advantage in giving more space in the interior. Like all cachets they require to be packed with care, and sliding boxes should be avoided. No druggist can be without some such apparatus, and we believe that this kind works to perfection.

---

**Tabloids: Guaiacum and Sulphur; Sulphonal; Aloin Compound; Dry Thyroid Gland Powder; Effervescing Cubebs and Belladonna; Arsenite of Copper; Pure Tar; Compound Cubeb; Iodic-Hydrarg.; Antipyrin, Sugar-coated; Soda Mint Granules, Sugar-coated.**—Burroughs, Wellcome and Co., London.—These are amongst the recent additions to the list of tabloids prepared by this well-known firm, whose name is a sufficient guarantee as to quality of drugs used and care in their preparation.

As regards the **Thyroid Gland Tabloids,** it is said that no extractive substances answer as well as the complete gland. The process of manufacture is thus described:—The glands are received three hours after the death of the sheep; they are supplied, at special request, attached to a piece of the trachea, from which they are carefully dissected and thinly sliced to discover whether any cysts or other morbid elements exist; they are then carefully dried *in vacuo* at a low temperature, and are pulverised and compressed together with a little common salt into "tabloids." Each "tabloid" represents five grains of the fresh lobe, and may be swallowed entire with a draught of fluid or administered in soup or other food.

The **Compound Cubeb Tabloids** contain two grains of powdered cubebs with chloride of ammonium and liquorice. For throat conditions and bronchial disorders they are likely to be especially useful at this season of the year.

---

**Emulsion of Petroleum, with Hypophosphites. Fluid Glymol. The Glymol Atomiser.**—Angier Chemical Company, Boston, U.S.A.—The **Emulsion** is an excellent preparation, containing hypophosphites of lime and soda, suspended in a seven-times purified hydrocarbon preservative. There can be no question as to the local uses of petroleum, and possibly it may serve some useful purpose in the alimentary canal, but we question if it can have any nutritive value and if it could reasonably be expected to take the place of an emulsion of cod liver oil. It appears to have obtained some reputation in the treatment of bronchitis, and has frequently been used when patients with phthisis have been unable to take cod liver oil in any form. It remains yet to be proved whether the petroleum emulsion is anything more than a well-prepared emulsion of hypophosphites; this it certainly is, and as such should have a considerable range of utility in many varieties of defective nutrition, either of nerve, muscle, or lung tissue.

The so-called **Fluid Glymol** is a limpid, colourless oil, which we have used for years as a basis for various hypodermic injections and oleaginous sprays.

The **Glymol Atomiser** is an excellent apparatus for producing these oleaginous sprays.

---

**Condensed Milk.**—ANGLO-SWISS CONDENSED MILK CO., London.—This Company's brands are said to be the original and the best, containing the most cream, amounting to from 10.63 to 11.95 per cent. Many other brands have been found to contain only from 1 to 3 per cent. of fatty matter. It is a thoroughly trustworthy milk, of uniform quality and of full nutritive value. Surely every child should thrive on such perfect nutriment as this.

---

**Milk of Magnesia. Palatable Cod Liver Oil Emulsion. Phospho-Muriate of Quinine Comp.**—THE CHAS. H. PHILLIPS CHEMICAL CO., New York.—These preparations have been forwarded to us by Messrs. Henry Hodder and Co., who are the agents for Bristol.

The **Milk of Magnesia** is simply a perfect magnesia hydrate: it is therefore a concentrated liquid magnesia, convenient in form, pleasant to take, and is a reliable antacid and aperient, four times the strength of the ordinary fluid magnesias now in use. It remains unchanged for any length of time, unless allowed to freeze. It may be given in doses of one to four teaspoonfuls.

The **Cod Liver Oil Emulsion** contains 50 per cent. of the oil, made into an excellent emulsion with wheat phosphates, mucilage, sugar, glycerine, pancreatine, and water. It must be a concentrated, easily digestible, and nutritious food.

The **Phospho-Muriate of Quinine** contains the wheat phosphates combined with quinine, iron, and strychnine.

---

**Meat Juice.**—BRAND AND CO., London.—This is said to be the juice of the finest English meat in its natural state, containing the albumen uncoagulated and ready for immediate assimilation. We have ascertained that it does contain a large amount of soluble albumen, and that it is as palatable as it must be nutritious. We fail to see why for purposes of this kind so much emphasis is given to the English origin of the meat.

---

**Thyroid Extract. Thyroid Powder. Thyroid Capsules.**—FERRIS & Co., Bristol.—These preparations, for the treatment of cases of myxœdema, have been devised with care, and may be relied upon as thoroughly active. This mode of administration is a great improvement on the feeding with uncooked gland or the hypodermic use of an emulsion of the gland. Sixty grains of the powder represent one fresh gland.

---

**Combined Match Box and Throat Lamp. Patent Aseptic Aluminium Syringe.**—BURROUGHS, WELLCOME AND CO., London.—The first of these articles is an ingenious little contrivance, designed by Mr. H. W. Denton Cardew, for readily illuminating the throat, nose, ear, or vagina. It is made of a white metal, which readily reflects the light of an ordinary wax match. It is sold at the cheap price of two shillings, and can be readily carried in the waistcoat pocket. We have tried it and found it very effectual, especially for looking at the tonsils and fauces.

The syringe is a distinct improvement on its predecessors, and appears to be well worthy of the highest award for syringes recently given at the Chicago Exhibition. It is available for all solutions requiring subcutaneous administration. It is not affected by any climatic conditions, and is fitted with a perfectly aseptic packing. The glass barrel is graduated on one side in minims indelibly marked in black, and on the other with metric scale—one cubic centimetre divided into millimetres—in red.

---

The litigation connected with Kutnow's improved Effervescent Carlsbad Powder has terminated in favour of Messrs. Kutnow and Co., who may now, in spite of the opposition of the Municipality of Carlsbad, use the picture of the rock surmounted by a chamois, with the words, "Hirschensprung, or Deer Leap, Carlsbad." Mr. Justice North decided all points in favour of the applicant, as showing that the effect of the trade mark was not likely to deceive the public into the belief that the applicant's powder was the natural as opposed to the artificial product. The *Lancet* points out that "for the future the name 'Carlsbad' when applied to powders is to be dissociated from any special geographical meaning, and is to be understood as indicating only the possession of certain medicinal effects not necessarily connected with the place."

